# Evaluating Exoskeletons for WMSD Prevention: A Systematic Review of Applications and Ergonomic Approach in Occupational Settings

**DOI:** 10.3390/ijerph21121695

**Published:** 2024-12-19

**Authors:** André Cardoso, Andreia Ribeiro, Paula Carneiro, Ana Colim

**Affiliations:** 1 DTx Digital Transformation Colab, Campus of Azurém, University of Minho, 4800-058 Guimarães, Portugal; andre.cardoso@dps.uminho.pt (A.C.);; 2 Algoritmi Research Centre/LASI, School of Engineering, University of Minho, 4800-058 Guimarães, Portugal; 3 Bosch Car Multimédia S.A., Manufacturing Engineering, 4705-820 Braga, Portugal; andreia.ribeiro@pt.bosch.com

**Keywords:** exoskeleton, WMSD, ergonomics, occupational settings, risk assessment methods

## Abstract

This review provides a comprehensive analysis of studies investigating the impact of occupational exoskeletons on work-related musculoskeletal disorder (WMSD) risk factors. The primary objective is to examine the methodologies used to assess the effectiveness of these devices across various occupational tasks. A systematic review was conducted following the PRISMA guidelines, covering studies published between 2014 and 2024. A total of 49 studies were included, identified through searches conducted in Scopus and Web of Science databases, with the search string launched in August 2024. The review identifies a growing body of research on passive and active exoskeletons, with a notable focus on laboratory-based evaluations. The results indicate that direct measurement and self-report methods are the preferred approaches in these domains. Ergonomic limitations and user discomfort remain concerns in some cases. The findings of this review may influence stakeholders by providing insights into the potential benefits of adopting exoskeletons and improving workplace ergonomics to reduce WMSD risks. Additionally, the identification of WMSD assessment methods will be valuable for validating the use of these technologies in the workplace. The review concludes with recommendations for future research, emphasizing the need for more real-world assessments and improved exoskeleton designs to enhance user comfort and efficacy.

## 1. Introduction

Work-related musculoskeletal disorders (WMSDs) have long been recognized as a leading cause of occupational injuries [1], accounting for a significant proportion of absenteeism, reduced work productivity, and long-term disabilities worldwide [2]. The European Agency for Safety and Health at Work (EU-OSHA) [3] reports that approximately 60% of workers across Europe are affected by musculoskeletal discomfort, underscoring the widespread impact of these conditions.

Scientific literature systematically indicates that WMSD prevalence is higher among specific working populations and occupational sectors compared to the general population [4]. This suggests a causal relationship between various occupational risk factors and the development of these conditions. Risk factors such as awkward body postures, repetitive movements, manual handling of heavy loads, mechanical vibrations, and work-related stress play a significant role in the development of WMSD [5]. Consequently, industries where these risk factors are prevalent, such as manufacturing, construction, and logistics, tend to report higher rates of WMSD [6]. These physical demands place substantial biomechanical stress on body areas such as the back, shoulders, and upper and lower limbs [7].

In recent years, the development and implementation of occupational exoskeletons have emerged as a potential solution to mitigate the risks associated with these physically demanding tasks [8]. The evidence supporting these claims is presented in this review’s Section 3, providing detailed insights into their effectiveness.

These exoskeletons are wearable devices designed to provide mechanical support to the body, helping to reduce the biomechanical strain on muscles and joints [9,10,11]. Specifically, exoskeletons are designed to limit muscle movement, reduce the effort required by the body to perform tasks, and/or help maintain non-neutral postures [12]. Typically made from materials like carbon fiber, aluminum, or plastic, these devices feature a lightweight frame or structure that can be worn on the torso, arms, or legs [13].

In addition to their general design, exoskeletons can be classified based on the body part they support [14]. Upper body exoskeletons assist the arms, shoulders, and torso, reducing fatigue and improving precision during tasks involving repetitive overhead motions or heavy lifting [15]. Lower body exoskeletons support the hips, legs, and knees, improving mobility and stability while reducing strain in activities involving standing or sitting postures [10]. Lower back exoskeletons are specifically designed to support the lumbar region, helping to prevent injuries and reduce strain during lifting, bending, or prolonged standing [16], making them particularly useful in industries where lower back pain is common. There are also exoskeletons developed to provide support to specific parts of the body. Their usefulness depends on the particular body part they are designed to support. For example, some exoskeletons are specifically designed to offer support to the elbow [17] or neck [18]. Furthermore, exoskeletons can also be classified based on their actuation as either active or passive [10]. Passive exoskeletons typically rely on spring-based mechanisms to redistribute the user’s body weight and reduce strain [19,20]. In contrast, active exoskeletons use motors or actuators to provide powered assistance [21], making tasks like lifting or overhead work easier by directly enhancing the user’s strength and endurance.

Studies have shown that exoskeletons can significantly reduce WMSD and perceived discomfort [22,23,24,25]. However, assessing their effectiveness in lowering WMSD risk is a complex and non-standardized process due to the wide range of ergonomic evaluation methods. These methods are typically divided into three categories: self-report and checklists, observational methods, and direct measurement techniques [26,27,28]. Self-reports and checklists are commonly used tools in occupational ergonomics [29], with validated questionnaires like the Nordic Musculoskeletal Questionnaire (NMQ) [30] used to gather workers’ perceptions and checklists such as the “OSHA checklist” from the Occupational and Health Administration (OSHA) [31] applied to identify risk factors. Observational methods, including tools like the Rapid Entire Body Assessment (REBA) [32] and the Key Indicator Method for Manual Handling Operations (KIM-MHO) [33], rely on direct observation of work tasks, assessing factors like task frequency, duration, and load handling to evaluate the external physical workload [27]. Direct measurement methods involve the use of sensors to assess the effect of risk factors on physiological and biomechanical parameters [26], with examples including surface electromyography (EMG) [34,35] to measure muscular activity and motion capture devices [36] to record joint motion.

The methodologies used to assess WMSD risk factors in exoskeleton studies vary considerably. This diversity in approaches makes it challenging to compare findings across different studies.

The methodologies used to assess WMSD risk factors in exoskeleton studies vary considerably. This diversity in approaches makes it challenging to compare findings across different studies. Within this scope, other review studies have primarily focused on specific aspects of exoskeleton use or their general impact on occupational health and safety, but often leave methodological considerations underexplored. For instance, Kranenborg et al. (2023) [37] focused on the side effects and usability of shoulder and back-support exoskeletons, primarily in laboratory settings, noting the lack of long-term and real-world assessments. Similarly, Flor-Unda et al. (2023) [12] reviewed the role of exoskeletons in reducing physical strain across various industries but lacked a detailed analysis of the methodologies used to assess their effectiveness in specific tasks. Kermavnar et al. (2021) [38] reviewed industrial back-support exoskeletons and emphasized the need for more field studies, as most of the research was conducted in controlled environments with healthy young men. These limitations underscore the need for a review that bridges these gaps by providing an examination of the methodologies used in the evaluation of both active and passive exoskeletons. This review uniquely addresses these gaps by exploring trends in the ergonomic evaluation methods applied. The major contribution of this paper is, to sum up studies that have focused on the evaluation of occupational exoskeleton, providing detailed information on their methodological approach and highlighting the current state of the art in this research field.

## 2. Materials and Methods

This review was guided by the methodology of the Preferred Reporting Items for Systematic Reviews and Meta-Analyses (PRISMA) [39]. Since its inception in 2009, the PRISMA methodology has provided a transformative framework for developing literature reviews, ensuring they are comprehensive, transparent, and unbiased [40]. The PRISMA-ScR checklist (Table A1 in Appendix A) was used in this review.

### 2.1. Information Source, Screening, and Eligibility Criteria

**Figure 1:**
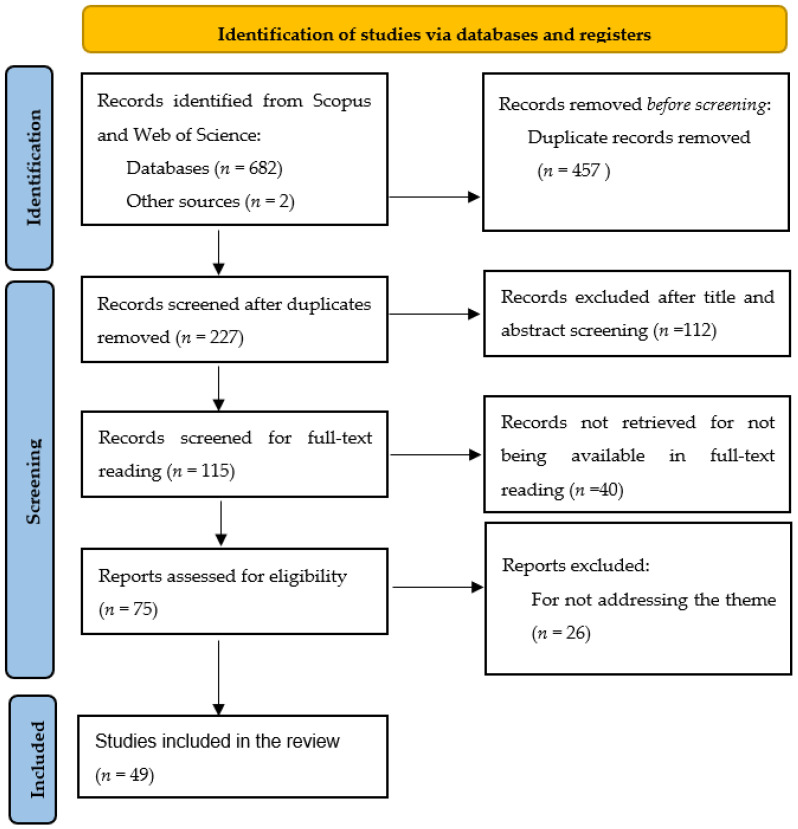
Steps of the PRISMA protocol for the literature review on WMSD risk assessment methods in assistive working (adapted from Moher et al. (2009) [39]).

First, a detailed search was performed using the Scopus and Web of Science databases. The selection of these databases was justified by their prominence and relevance to publications in the engineering and manufacturing domains [41]. Additionally, the decision to limit our search to only two databases align with practices in other systematic reviews that have adopted the PRISMA methodology [37,38], particularly in fields where these databases provide comprehensive coverage of the relevant literature. The search strategy included the keywords “Exoskeleton” and “WMSD” and focused on articles published between 2014 and 2024. This timeframe was chosen to reflect recent developments in occupational exoskeleton research, which has gained significant attention in the last decade. The initial search retrieved 682 articles, and an additional two articles from a personal database were included, resulting in a total of 684 records. The steps undertaken to conduct the systematic review are presented in Figure 1.

The screening phase aimed to filter the articles based on predefined inclusion criteria. Only papers written in English, available as open-access publications, and provided as full-text journal articles or conference papers were considered. To manage the dataset, Microsoft Excel Version 16.03 was used to organize and sort the records by title, facilitating the removal of duplicates. This step reduced the dataset to 227 studies for further evaluation. Then, titles and abstracts were screened to identify the studies specifically related to occupational exoskeletons. To be included, articles had to meet several criteria. They needed to involve healthy adults within the working age range and focus on exoskeletons designed to reduce physical load. Studies were required to take place in a workplace environment or a laboratory setting explicitly described as simulating or imitating workplace conditions. Furthermore, the application of WMSD risk assessment methods was mandatory, and only studies with full-text availability were considered.

Studies included in the review either had a control group, including a control group without exoskeleton use, or compared different exoskeleton types. Articles were excluded if they did not assess WMSD risk using ergonomic assessment methods, focused on non-occupational exoskeleton applications such as rehabilitation or military purposes, or involved participants outside the working-age population. By narrowing the scope to only include occupational applications, we aim to ensure that the review provides a targeted and in-depth analysis of the methodologies and effectiveness of exoskeletons in the specific context of workplace use. No restrictions were applied regarding study design, allowing for a comprehensive evaluation of the existing literature.

Finally, full-text analysis was conducted on 115 eligible studies to ensure they aligned with the objectives of the review. Of these, 40 were not available for full-text reading, leaving 75 studies available for analysis. From these, 26 were excluded for not meeting the inclusion criteria, resulting in 49 articles included for review. This systematic approach provided a robust foundation for examining the impact of occupational exoskeletons on WMSD risk factors and their broader implications for workplace ergonomics.

### 2.2. Data Extraction and Analysis

After the selection and full-text reading of the articles to be included, a table was constructed in Microsoft Excel Version 16.03 to systematically extract and organize the relevant information from each study. The primary aim was to gather key details to better understand the methods employed and inform future research. The extracted information included the following: authorship details, publication year, number of participants, type of exoskeleton tested (passive or active), body part supported by the exoskeleton, study context (whether the study was conducted in a laboratory, simulated on-site, or on-site), types of tasks studied, WMSD risk assessment methods applied, and the main conclusions drawn from each study.

Subsequently, additional tables and graphs were created to more effectively visualize the extracted data, enabling a clearer interpretation and comparison of findings. This structured and graphical approach facilitated a comprehensive analysis of the methodologies and outcomes in the field of occupational exoskeletons and WMSD risk assessment.

## 3. Results and Discussion

This section presents the findings of the systematic review, organized into four key areas. First, an overview of the reviewed studies is provided, including the publication years, participant demographics, and study contexts (Section 3.1). Next, the focus shifts to the exoskeletons examined, detailing their design features, actuation types, and supported body parts (Section 3.2). The methodological approaches employed in the studies are then discussed, highlighting the diversity of WMSD risk assessment methods and their applications (Section 3.3). Finally, the main conclusions of the reviewed studies are summarized, offering insights into the effectiveness, challenges, and future potential of occupational exoskeletons (Section 3.4).

### 3.1. Overview of the Year, Participants, and Study Context

To provide a broader perspective on the included studies, an overview of the publication year, number of participants, and study context is first presented (Table 1). It should be noted that the number of participants is also segmented by gender, with the average age, height, and weight being presented, when available.

Related to the years of publication distribution, the studies on the evaluation of occupational exoskeletons show a clear upward trend over the years. Beginning with a minimal number of publications in 2018 [42] and 2019 [43], each with just one study, there is a gradual increase in research output. In 2020 [9,24,44], and 2021 [22,23,45,46] the number of studies rose to four, followed by a jump to seven published in 2022 [11,47,48,49,50,51,52,53].

A substantial surge in research activity was observed in 2023 [16,17,18,21,25,54,55,56,57,58,59,60,61,62,63,64,65,66,67,68], with 20 studies being published, reflecting a peak in scholarly interest in the field. While there was a slight decline in 2024 [10,14,15,20,69,70,71,72,73,74,75], with 11 studies, the overall trend demonstrates a sustained and growing interest in the evaluation of occupational exoskeletons over this period. Notably, no study was found between the period of 2014 to 2017.

**Table 1:** Articles distribution according to the number of participants and study context.

Authors (Year)	Number of Participants	Study Context
Moyon et al. (2018) [42]	*n* = 9 (♂5; ♀4); Age: 20–40, Height N.A., Weight: N.A.	On-site
Schmalz et al. (2019) [43]	*n* = 12 (♂6; ♀6); Age: 24.0, Height 176.0 cm, Weight: 73.0 kg	Laboratory
Perez Luque et al. (2020) [9]	*n* = 17 (♂11; ♀6); Age: 25, Height 174.0, Weight: N.A.	Simulated On-site
Alabdulkarim et al. (2020) [44]	*n* = 16 (♂16; ♀0); Age: 34.3 Height 164.4 cm, Weight: 70.0 kg	Laboratory
Cardoso et al. (2020) [76]	*n* = 5 (♂2; ♀3); Age: 29.0, Height 165.0 cm, Weight: 76.0 kg	On-site
Lazzaroni et al. (2020) [24]	*n* = 9 (♂9; ♀0); Age: 27.3, Height 182.0 cm, Weight: 73.8 kg	Laboratory
Kong et al. (2021) [23]	*n* = 20 (♂13 Age: 22.5, Height 176.4 cm, Weight: 72.0 kg; ♀7); Age: 20.7, Height 165.5 cm, Weight: 57.2 kg)	Laboratory
Schwartz et al. (2021) [45]	*n* = 29 (♂15 Age: 23.0, Height 179.0 cm, Weight: 77.0 Kg; ♀14 Age: 22.0, Height 167.0 cm, Weight: 58.0 kg)	Laboratory
Lazzaroni et al. (2021) [46]	*n* = 10 (♂10; ♀0); Age: 29.8, Height 177.8 cm, Weight: 74.4 kg	Laboratory
Yin et al. (2021) [22]	*n* = 10 (♂10; ♀0); Age: 24.7, Height 174.8 cm, Weight: 68.3 kg	Laboratory
Weston et al. (2022) [47]	*n* = 6 (♂10 Age: 21.2, Height 179.5 cm, Weight: 79.8 kg; ♀6 Age: 22.5, Height 165.5 cm, Weight: 57.6.4 kg)	Laboratory
vam der Have et al. (2022) [48]	*n* = 16 (♂8; ♀8); Age: 21.9, Height N.A., Weight: N.A.	Laboratory
Kong et al. (2022) [49]	*n* = 20 (♂20; ♀0); Age: 24.8, Height 176.4 cm, Weight: 78.8 kg	Laboratory
Iranzo et al. (2022) [50]	*n* = 8 (♂4; ♀4); Age: 35.0, Height 175.6 cm, Weight: 67.9 kg	Laboratory
Latella et al. (2022) [11]	*n* = 12 (♂12; ♀0); Age: 23.2, Height 179.3 cm, Weight: 72.7 kg	Laboratory
De Bock et al. (2022) [51]	*n* = 22 (♂22; ♀0); Age: 23.7, Height 181.6 cm, Weight: 75.9 kg	Laboratory
Goršič et al. (2022) [52]	*n* = 10 (♂5; ♀5); Age: 28.4, Height 170.0 cm, Weight: 71.2 kg	Laboratory
Sierotowicz et al. (2022) [53]	*n* = 12 (♂9; ♀3); Age: 27.6, Height 176.0 cm, Weight: 71.9 kg	Laboratory
Mitterlehner et al. (2023) [54]	*n* = 30 (♂22; ♀8); Age: 29.0, Height 180.2 cm, Weight: 74.8 kg	Laboratory
R. M. Van Sluijs et al. (2023) [55]	*n* = 14 (♂5; ♀9); Age: 25.3, Height 170.0 cm, Weight: 70.7 kg	Laboratory
Garosi et al. (2023) [18]	*n* = 14 (♂14; ♀0); Age: 28, Height 176.0 cm, Weight: 71.6 kg	Laboratory
Walter et al. (2023) [56]	*n* = 14 (♂11; ♀3); Age: 22.3, Height 177.7 cm, Weight: 71.9 kg	Laboratory
Kong et al. (2023) [57]	*n* = 20 (♂20; ♀0); Age: 24.4, Height 176.0 cm, Weight: 78.0 kg	Laboratory
Shim et al. (2023) [58]	*n* = 20 (♂20; ♀0); Age: 24.4, Height 176.0 cm, Weight: 78.0 kg	Laboratory
Brunner et al. (2023) [59]	*n* = 32 (♂17; ♀15); Age: 26.7, Height 174.0 cm, Weight: 72.1 kg	Laboratory
Öçal et al. (2023) [21]	*n* = 3 (♂2; ♀1); Age: 33.3, Height 179.0 cm, Weight: 71.6 kg	Laboratory
Schrøder Jakobsen et al. (2023) [61]	Control: *n* = 10 (♂N.A.; ♀N.A.); Age: 32.2, Height 180.3 cm, Weight: 82.4 kg; Intervention n = 10 (♂N.A.; ♀N.A.); Age: 33.3, Height 181.9 cm, Weight: 87.4 kg	On-site
Govaerts et al. (2023) [60]	*n* = 16 (♂10; ♀6); Age: 35.0, Height 173.9 cm, Weight: 72.4 kg	Laboratory
Verdel et al. (2023) [62]	*n* = 19 (♂12; ♀7); Age: 24.0, Height 173.0 cm, Weight: 66.7 kg	Laboratory
Reimeir et al. (2023) [63]	*n* = 12 (♂9; ♀3); Age: 27.2, Height 179.4 cm, Weight: 75.3 kg	Laboratory
R.M. van Sluijs et al. (2023) [16]	*n* = 30 (♂22; ♀8); Age: 27.0, Height 178.0 cm, Weight: 72.9 kg	Laboratory
Park et al. (2023) [17]	*n* = 5 (♂3; ♀2); Age: 28.8, Height 175.0 cm, Weight: 65.4 kg	Laboratory
Ding et al. (2023) [25]	*n* = 9 (♂9; ♀0); Age: 24.6, Height 176.3 cm, Weight: 72.2 kg	Laboratory
Cuttilan et al. (2023) [64]	*n* = 10 (♂N.A.; ♀N.A.); Age: N.A., Height 170.0 cm, Weight: N.A.	Laboratory
De Bock et al. (2023) [65]	*n* = 16 (♂16; ♀0); Age: 29.3, Height 181.0 cm, Weight: 81.4.5 kg	Laboratory
Bhardwaj et al. (2023) [66]	*n* = 10 (♂16; ♀0); Age: 21–28, Height 171.1 cm, Weight: 71.2 kg	Laboratory
Thang (2023) [67]	*n* = 10 (♂10; ♀0); Age: 18–22, Height 170.0 cm, Weight: 70 kg	Laboratory
Schwartz et al. (2023) [68]	*n* = 29 (♂15 Age: 25.0, Height 180.0 cm, Weight: 74.9.kg; ♀0); Age: 24.0, Height 166.0 cm, Weight: 63.6.5 kg	Laboratory
Musso et al. (2024) [14]	*n* = 18 (♂18; ♀0); Age: 27.11, Height 179.5 cm, Weight: 78.67 kg	Laboratory
Schrøder Jakobsen et al. (2024) [69]	Control: *n* = 10 (♂7; ♀3); Age: 30.3, Height 177.9 cm, Weight: 81.1 kg; Intervention *n* = 9 (♂7; ♀2); Age: 29.8, Height 181.0 cm, Weight: 81.8 kg	On-site
Rafique et al. (2024) [10]	*n* = 9 (♂7; ♀2); Age: 30.0, Height 160.0–185.0 cm, Weight: 160.0–180.0 kg	Laboratory
Davoudi Kakhki et al. (2024) [70]	*n* = 22 (♂10; ♀12); Age: 20.5, Height N.A., Weight: 66.3 kg	Laboratory
van Sluijs et al. (2024) [71]	*n =* 31 (♂16; ♀15); Age: 28.0, Height 176.3 cm, Weight: 76.4 kg	Laboratory
Favennec et al. (2024) [72]	*n* = 18 (♂18; ♀0); Age: 21.5, Height 178.3 cm, Weight: 69.6 kg	Laboratory
Lee et al. (2024) [73]	*n* = 5 (♂5; ♀0); Age: 27.0, Height 174.8 cm, Weight: 70.0 kg	Laboratory
Gräf et al. (2024) [15]	*n* = 10 (♂5; ♀5); Age: 25.3, Height 174.6 cm, Weight: N.A.	Laboratory
Govaerts et al. (2024) [74]	*n* = 18 (♂10; ♀8); Age: 33.0, Height 173.5 cm, Weight: 70.6 kg	Laboratory
Bär et al. (2024) [75]	*n* = 36 (♂36; ♀0); Age: 25.9, Height 178.7 cm, Weight: 73.5 kg	Laboratory
Cardoso et al. (2024) [20]	*n* = 2 (♂1; ♀1); Age: 25.5, Height 176.0 cm, Weight: 77.5 kg	Simulated On-site

This progression suggests an expanding recognition of the importance and relevance of exoskeletons in occupational settings, as well as a corresponding increase in academic and practical investigation into their applications and impacts.

The distribution of the number of participants per study in the evaluation of occupational exoskeletons reveals a varied approach to sample sizes across the reviewed literature. The data indicates a preference for certain participant group sizes, with the most common being studies involving 10 participants [15,22,46,52,61,64,66,67,69], as reflected by the fact that nine studies employed this number. This is followed by studies with 9 [10,25,42,46], 12 [11,43,53,63], 16 [44,48,60,65], and 20 participants [23,49,57,58], each represented in four studies.

Smaller sample sizes, such as 5 participants, were utilized in three studies [17,73,76], while studies with more participants, namely 14 [16,18,56], and 18 [14,72,74] also appeared three times. Several other studies employed sample sizes ranging from 2 to 36 participants, but these were less common and typically involved only one or two studies per group size. Notably, the studies with the highest number of participants, 31 [71], 32 [59], and 36 [75], were each represented by a single study.

This distribution suggests that while there is no standardized sample size for evaluating occupational exoskeletons, there is a slight tendency towards moderate-sized groups, with 10 participants being particularly favored. The variance in participant numbers could reflect the diversity of research designs, objectives, and resource availability within the field.

The distribution of study contexts in the evaluation of occupational exoskeletons reveals a strong preference for laboratory-based research. According to the data, 43 studies were conducted in a laboratory setting [10,11,14,15,16,17,18,21,22,23,24,25,43,44,45,46,47,48,49,50,51,52,53,54,55,56,57,58,59,60,62,63,64,65,66,67,68,70,71,72,73,74,75], indicating that controlled environments are the predominant choice for examining the efficacy and safety of exoskeletons.

In contrast, only a small number of studies—four in total—were carried out on-site [42,61,69,76], directly within the work environment. Even fewer studies—just two—employed a simulated on-site context [9,20], where real-world conditions were mimicked but not conducted at the actual worksite.

This distribution suggests that while laboratory settings offer the advantages of control and repeatability, there is a growing, albeit limited, interest in evaluating exoskeletons under more realistic conditions, either through on-site testing or simulations. However, the relatively low number of studies in real-world settings highlights a potential area for further research to better understand how these devices perform in practical, everyday use.

### 3.2. Overview of the Exoskeletons Studied

This subchapter provides a comprehensive overview of the exoskeletons examined in the reviewed studies, highlighting their mode of actuation (active or passive) and the specific body regions they are intended to support. This information is crucial for understanding the diverse applications and technological approaches of exoskeletons in occupational settings. Table 2 summarizes the key characteristics of the exoskeletons featured in these studies, offering a concise yet comprehensive view of the current state of research in this domain.

The data indicates that most of the reviewed studies focused on evaluating a single exoskeleton, with 38 studies [11,14,15,16,17,18,21,22,23,24,25,42,43,44,46,48,49,50,51,52,53,54,55,56,59,61,62,64,65,66,67,69,70,71,72,73,75,76] adopting this approach. In contrast, 11 studies [9,10,20,45,47,57,58,60,63,68,74] examined more than one exoskeleton, suggesting a comparative analysis of different devices within those studies. This distribution reflects a prevalent research trend where a singular exoskeleton is typically selected for in-depth evaluation, possibly due to resource constraints or a desire to thoroughly investigate the specific features and performance of individual devices. However, the presence of studies that assess multiple exoskeletons highlights an emerging interest in comparing the effectiveness, usability, and suitability of various exoskeleton models within occupational settings. This comparative approach could provide valuable insights into the relative advantages and limitations of different technologies, guiding future design and application in the field.

The data on the exoskeletons studied in the reviewed literature reveals a diverse range of devices, with some exoskeletons being more frequently evaluated than others. Among the exoskeletons, the Paexo [9,11,43,53,54,60,74] is the most frequently studied, appearing in seven different studies. This is followed by Cray X [56,60,63,68,74], which is featured in five studies. Several exoskeletons, including BackX [68,69,70] Auxivo lift suit V2 [16,20,55], Cex [23,49,58], and Laevo V2 [50,75,76], were each assessed in three studies, indicating a moderate level of research interest.

Some exoskeletons, such as Airframe [47,57], BionicBack hTRIUS [20,63], Corfor [45,72], EksoVest [10,47], Shoulder X [47,61], Shoulder exoskeleton prototype (Exo4Work) [51,65], Skelex X [14,15], and XoTrunk [24,46], were each studied in two different investigations, reflecting ongoing but less concentrated research attention.

Most of the exoskeletons listed were studied in only one investigation, underscoring the broad exploration of different exoskeleton designs within the field. These single-study devices include prototypes and specialized models, such as the OmniSuit [71], Pad [42], Elbow-sideWINDER [17], and Head/neck supporting exoskeleton [18].

This distribution highlights the focus on certain exoskeletons that are either more commercially available or have shown promise in early studies, while also reflecting the experimental nature of many of the devices being evaluated. The variation in the frequency of studies on specific exoskeletons may be influenced by factors such as availability, intended use cases, and the specific ergonomic or functional challenges they address.

The studies of the type of actuation in the exoskeletons reveal a preference for passive exoskeletons. Of the exoskeletons reviewed, 56 are passive [9,10,11,14,15,16,18,20,21,22,23,25,42,43,44,45,47,48,49,50,51,52,53,54,55,56,57,58,59,60,61,62,63,64,65,66,67,68,69,70,71,72,73,74,75,76], while only 11 [17,24,46,56,60,62,63,68,74] are active. This disparity suggests that most research in occupational exoskeletons has focused on passive systems, which do not require external power sources and typically rely on mechanical structures to support or redistribute the user’s physical load [77].

The preference for passive exoskeletons may be driven by several factors, including their simpler design, lower cost, and potentially easier integration into various work environments. Passive systems are often more practical in settings where minimal maintenance and operational simplicity are crucial.

In contrast, active exoskeletons, powered by motors or actuators, potentially providing more dynamic assistance [77], appear less frequently in the studies. This may reflect the higher complexity, cost, and potential technical challenges associated with active systems, although they can offer greater versatility and support in certain applications.

Overall, the data indicates a predominant focus on passive exoskeletons in the current literature, with active systems representing a smaller, yet significant, area of interest. This trend may evolve as technology advances and the potential benefits of active exoskeletons become more accessible.

The body parts supported by the exoskeletons in the reviewed studies show a clear focus on devices designed to assist the back and upper arms. Specifically, 32 exoskeletons are intended to support the back [16,20,24,25,45,46,50,52,54,56,59,60,63,64,66,69,70,72,74,75,76], making it the most targeted area. This emphasis likely reflects the high incidence of back-related injuries in occupational settings [78], where lifting and repetitive movements are common [79].

Following closely, 26 exoskeletons provide support for the upper arm [9,10,11,14,15,21,22,42,43,47,48,51,53,57,59,61,62,65,67,73], which is also a critical area in many manual labor tasks that involve overhead work or heavy lifting [80]. A smaller subset of devices, two exoskeletons [44,71], supports both the upper arm and back, suggesting an integrated approach to reducing strain on both areas simultaneously.

Other body parts are less frequently addressed. Five exoskeletons are designed to support the legs [10,23,49,58], likely for tasks that involve prolonged standing or heavy leg movement. The head and neck [18] and the elbow [17] are the least supported areas, with only one exoskeleton dedicated to each. This indicates that while these regions may also be vulnerable to strain, they are not the primary focus of current exoskeleton technology in occupational settings.

The distribution reflects the prioritization of support for the back and upper arms, which are the most susceptible to injury in many physically demanding workplaces. This trend aligns with the broader goals of occupational exoskeletons to prevent musculoskeletal disorders and enhance worker safety and efficiency.

### 3.3. Overview of the Methodological Approach

In this section, a comprehensive summary of the methodological strategies employed in the reviewed studies is provided, as presented in Table 3. This table outlines the types of occupational tasks that were analyzed, reflecting the diversity of scenarios in which exoskeletons have been evaluated. Furthermore, the table details the WMSD risk assessment methods utilized across the studies. These methods are categorized into three primary groups: direct measurement, which is further divided into biomechanical and physiological measurements; observational methods; and self-reports and checklists. This segmentation offers a structured view of the approaches taken to assess the effectiveness and ergonomic impact of exoskeletons in mitigating the WMSD risk.

The data regarding the types of tasks evaluated in studies on occupational exoskeletons highlights a varied focus on different physical activities. The most frequently assessed tasks involve a combination of different tasks, with 17 studies adopting this approach. This suggests a comprehensive evaluation strategy where exoskeletons are tested across multiple task types to determine their overall effectiveness and versatility in various work scenarios.

Lifting tasks are the next most studied, featured in 12 studies. This focus reflects the significant role that lifting plays in many occupational settings and the associated risk of musculoskeletal disorders, particularly in the back and upper limbs. Similarly, overhead tasks are a central focus, with 11 studies examining exoskeletons’ ability to support activities that involve reaching or working above shoulder level—a common cause of strain in industrial and construction work.

Other tasks such as forward leaning and reaching tasks are each addressed in three studies, indicating a moderate interest in evaluating exoskeletons’ effectiveness in supporting these specific movements. Tasks performed at lower heights are also covered by three studies, which may include activities like squatting or kneeling, where exoskeletons could alleviate stress on the lower back and legs.

Finally, pushing/pulling tasks are the least frequently studied, with only two studies focusing on these activities. This suggests that while important, pushing and pulling may be considered less critical or more challenging to address with current exoskeleton designs compared to lifting or overhead tasks.

The data demonstrate a strong emphasis on evaluating exoskeletons in scenarios that pose significant ergonomic challenges, particularly those involving lifting and overhead work, while also recognizing the importance of multi-task assessments to gauge the devices’ practical applicability in diverse work environments. It is important to note that the distribution of the types of tasks extensively studied may be closely related to the specific type of exoskeleton being examined, particularly in terms of the body part it is designed to support.

Related to the WMSD risk assessment methods, the results reveal that only nine studies [10,15,18,21,22,25,46,53,73] adopted a single-method approach. While less common, this approach might have been chosen for its simplicity, focus, or resource constraints. In contrast, most of the studies applied a multi-method approach in their methodological design. This approach likely reflects the complexity and multifaceted nature of evaluating exoskeletons, as it allows for a more comprehensive assessment by combining various methods to capture different dimensions of their impact on WMSD. By utilizing multiple methods, researchers can triangulate their findings, increasing the robustness and reliability of the results [81].

We will now delve into each level of the WMSD risk assessment methods applied in the reviewed studies. Starting with direct measurement methods, focusing initially on those related to biomechanics, followed by an examination of physiological measurements.

**Figure 2:**
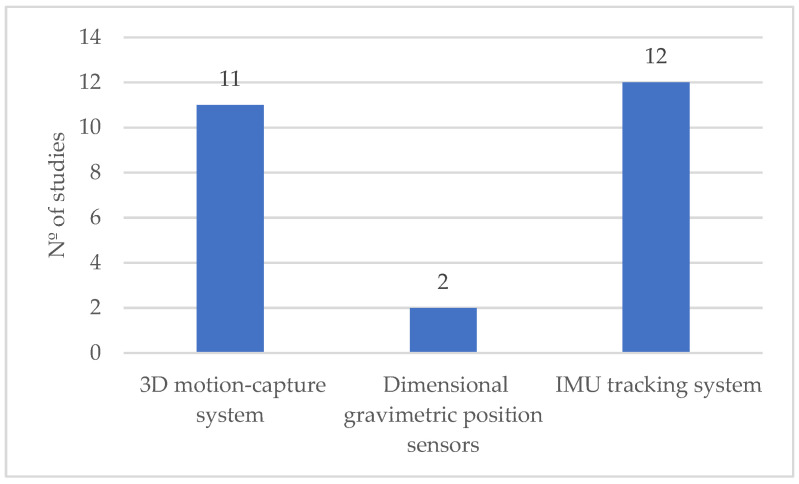
Distribution of the biomechanical direct measurement approaches by the number of studies.

The findings indicate that kinematic measurements were the predominant biomechanical method used in the reviewed studies (Figure 2), with 24 studies employing this approach [9,11,16,17,24,43,45,47,48,49,50,52,55,60,61,62,63,64,65,67,68,69,72,75]. In contrast, force measurements were much less commonly applied, being used in only two studies [11,49]. This suggests a preference for analyzing movement patterns and body mechanics (kinematics) over direct force exertion measurements in the context of WMSD risk assessment related to occupational exoskeletons. Given the previous observation that kinematics was the predominant biomechanical method used, the data further reveals that IMU tracking systems were the most employed kinematic measurement tools, utilized in 12 studies [9,11,17,45,49,50,55,61,63,67,68,69]. Close behind, 3D motion capture systems were also frequently used, appearing in 11 studies [16,24,43,47,48,52,60,62,64,65,72]. In contrast, dimensional gravimetric position sensors were employed in only one study [75]. This trend highlights a strong reliance on IMU tracking systems and 3D motion capture for capturing detailed movement data in studies assessing WMSD risks associated with occupational exoskeletons.

**Figure 3:**
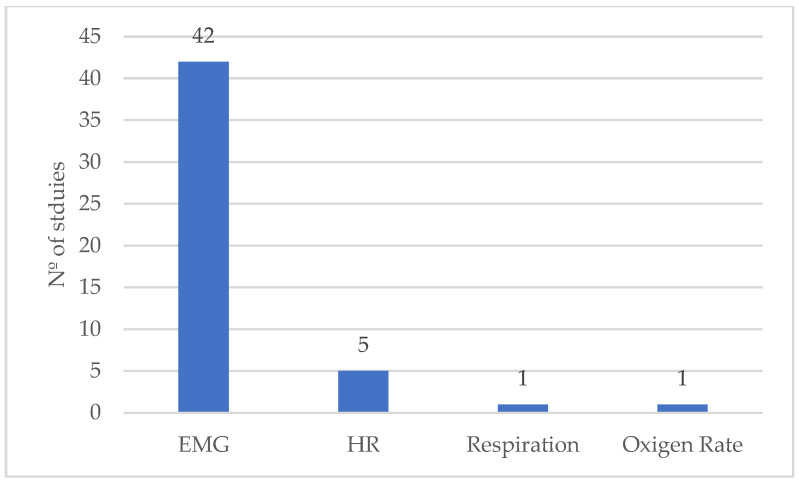
Distribution of the physiological direct measurement approaches by the number of studies.

Related to physiological direct measurement methods (Figure 3), EMG was the most used technique, appearing in four studies [10,14,15,16,17,18,20,21,22,23,24,25,43,44,45,46,47,48,49,50,51,52,53,55,56,57,58,59,61,62,63,64,65,66,67,68,69,71,72,73,75,76], indicating its critical role in assessing muscle activity during the use of occupational exoskeletons. In contrast, other physiological measures were far less prevalent, with heart rate (HR) being utilized in only five studies [42,51,54,59,75], and respiration [51] and the oxygen rate [43] each appearing in just one study. This disparity underscores the emphasis on EMG as the primary method for physiological assessment, while other metrics were used sparingly.

**Figure 4:**
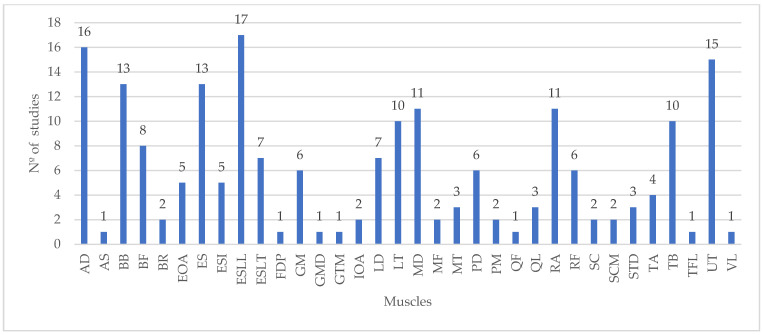
Distribution of the muscles studied by the number of studies. Anterior deltoid (AD); anterior serratus (AS); biceps brachii (BB); biceps femoris (BF); brachioradialis (BR); external obliquus abdominis (EOA); erector spinae (ES); erector spinae iliocostalis (ESI); erector spinae longissimus lumborum (ESLL); erector spinae longissimus thoracis (ESLT); flexor digitorum profundus (FDP); gluteus maximus (GM); gluteus medius (GMD); gastrocnemius medialis (GTM); internal obliquus abdominis (IOA); latissimus dorsi (LD); lower trapezius (LT); middle deltoid (MD); multifidus (MF); middle trapezius (MT); posterior deltoid (PD); pectoralis major (PM); quadriceps femoris (QF); quadratus lumborum (QL); rectus abdominis (RA); rectus femoris (RF); splenius capitis (SC); sternocleidomastoid (SCM); semitendinosus (STD); tibialis anterior (TA); tensor fascia latae (TFL); triceps brachii (TB); upper trapezius (UT); vastus lateralis (VL).

The analysis of the muscles studied through EMG in the reviewed literature is vital, as it not only informs future research on muscle selection for occupational exoskeleton studies but also enables the comparison of results across different studies. Therefore, Figure 4 presents the distribution of the muscles studied by the number of studies reviewed.

Among the muscles, the Erector Spinae Longissimus lumborum (ESLL), a key muscle for maintaining posture and supporting the lower back, was the most frequently studied, appeared in 16 studies [15,16,20,24,25,46,47,49,51,59,61,65,66,67,71,76]. This trend highlights the importance of monitoring back muscles, particularly in tasks that place significant strain on the lower back, a common area of concern in occupational settings.

The anterior deltoid (AD), found in 15 studies [14,15,18,21,22,44,48,51,53,55,59,61,62,71,73], and the Erector Spinae (ES), present in 13 studies [22,23,44,45,50,52,57,58,59,63,72,73,75], were also frequently analyzed. The anterior deltoid plays a crucial role in shoulder flexion [35], especially in overhead tasks, which are common in industrial environments. The consistent focus on these muscles suggests a priority in understanding how exoskeletons can alleviate stress in these areas. It is important to highlight that the attribution of ES in the aforementioned studies is quite broad, with no specification as to which particular muscle of the erector spinae group is being referred to. However, these data reinforce the previously demonstrated trend of focusing on back muscles in studies evaluating occupational exoskeletons.

Other superficial muscles, such as the biceps brachii (BB) [17,21,22,23,43,44,48,49,51,57,58,59,62], triceps brachii (TB) [17,21,22,23,49,51,57,58,59,62], and upper trapezius (UT) [14,18,21,23,43,49,51,57,58,61,67,69,73,76], were also frequently studied, each appearing in 10 to 14 studies. These muscles are involved in a variety of arm movements [82], including lifting and carrying tasks, emphasizing their significance in studies focused on upper limb support.

Notably, some muscles, such as the anterior serratus (AS) [43], gluteus medius (GMD), and quadriceps femoris (QF) [50] were studied in only a single study each. These muscles, despite their importance in stabilizing the shoulder, pelvis, and knee, respectively, have been underrepresented. Future research could expand on these muscles to gain a more comprehensive understanding of how exoskeletons interact with the entire musculoskeletal system.

The overall trend in muscle selection reflects a focus on superficial muscles, which are more accessible for EMG measurement and are directly involved in common occupational tasks. However, the underrepresentation of certain muscles suggests opportunities for future studies to explore these less-studied areas, enhancing our understanding of exoskeleton performance and its effects on a broader range of muscle groups.

This analysis is crucial for guiding future research, providing a foundation for selecting muscles to study in upcoming investigations and facilitating the comparison of results across studies. By expanding the focus to include a wider variety of muscles, researchers can develop a more holistic understanding of exoskeletons’ impacts on workers’ musculoskeletal health.

Regarding the observational methods, only one study applied the Rapid Entire Body Assessment (REBA) [20]. This reveals a significant gap in the use of observational tools within the reviewed literature. The limited application of REBA suggests that researchers may be prioritizing other assessment methods, such as direct measurements or self-reports, over observational techniques. This underutilization of observational methods highlights a potential area for future research, as these tools can provide valuable insights into the WMSD risks associated with exoskeleton use, particularly in complex or dynamic work environments.

**Figure 5:**
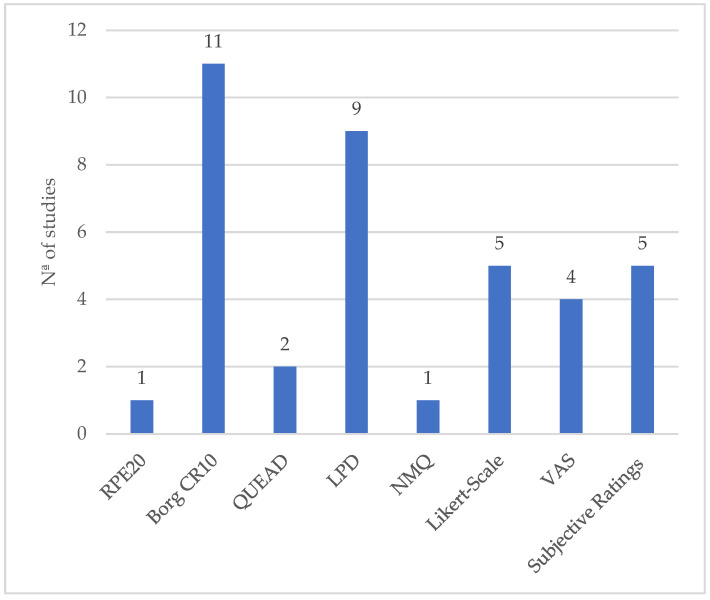
Distribution of the self-report/checklist methods by number of studies.

The analysis of self-reports and checklist methods distribution (Figure 5) reveals distinct trends in their application across the studies. These methods are critical for capturing subjective perceptions related to discomfort, pain, and overall workload, providing insights that complement objective measurements [26]. 

The Borg Category Ratio-10 Scale (Borg CR10) emerges as the most frequently utilized tool, with 11 studies employing it [20,23,42,44,57,59,61,67,69,70,76]. This scale is designed to assess perceived exertion, especially during physically demanding tasks [83]. Its frequent use demonstrates its effectiveness in assessing how participants perceive the effort involved in using exoskeletons compared to not wearing them or when comparing different exoskeleton devices. The Local Perceived Discomfort (LPD) scale was utilized in nine studies [10,16,17,21,51,52,60,61,76], indicating a strong focus on evaluating the specific discomforts experienced by participants in localized body regions, which is particularly relevant in studies involving exoskeletons where targeted muscle groups or joints may be affected.

Subjective ratings were employed in five studies, as well as the Likert scale. These tools are flexible and widely applicable, allowing researchers to measure various subjective experiences. In the case of the reviewed studies that employed these two metrics, the focus was on evaluating the following parameters: comfort/discomfort [9,47,62,67,69,71], range of motion [9,62], acceptance [67,69], ease of use [64,70], lift assistance [9], accuracy [62], usability [16,70], task difficulty, design [64], exertion, and perceived pressure [72].

The Visual Analog Scale (VAS) was used in four studies [54,66,74,76], indicating its utility in quantifying pain levels on a continuum, offering a simple yet effective way to capture variations in pain perception.

The Questionnaire for the Evaluation of Physical Assistive Devices (QUEAD) and the Nordic Musculoskeletal Questionnaire (NMQ) appeared in two [61,69] and one [54] studies, respectively. These tools are more specialized, with QUEAD focusing on examining usability, ease of use, comfort, and acceptance [84], abs NMQ on musculoskeletal symptoms [30], indicating their targeted use in particular study contexts.

Finally, the Ratio Perceived Exertion 20 (RPE20) scale was utilized in only one study [56], suggesting that it might be less commonly preferred for assessing perceived exertion in exoskeleton research, where other scales like Borg CR10 are more prominent.

This distribution highlights the varied approaches researchers take when assessing subjective outcomes in exoskeleton studies, with a clear preference for established methods like the Borg CR10 and LPD. These data provide a useful foundation for future studies, offering guidance on the selection of appropriate subjective assessment tools and facilitating the comparison of results across studies.

Globally, it is important to note that no study evaluating occupational exoskeletons relied solely on self-report or checklist methods, although some studies exclusively applied direct measurement methods. In most cases, a multimethod approach was used, combining direct measurements and self-report methods. Direct methods provide objective data that accurately reflect the exoskeleton’s performance in occupational settings. However, they can be resource intensive and may not capture the subjective experience of the wearer. On the other hand, self-report and checklist methods offer valuable insights into user perceptions, comfort, and usability, but they are inherently subjective and may be influenced by biases or inaccuracies.

The combination of both methods addresses the limitations of each approach. By integrating direct measurements with subjective assessments, a more comprehensive evaluation may be achieved, providing both objective data and a deeper understanding of user experience. The multimethod approach is essential in assessing WMSD risk factors [85] and therefore in evaluating the effectiveness of occupational exoskeletons in a holistic manner.

### 3.4. Overview of the Main Conclusions of the Reviewed Studies

The synthesis of various studies focused on the effectiveness of passive and active exoskeletons in reducing physical strain and preventing WMSD across different industrial tasks is presented in Table 4. The objective and main conclusions are presented. The studies collectively assess the impact of exoskeletons on muscle activity, discomfort, physical workload, and task performance in occupations that involve repetitive or physically demanding activities, such as overhead work, lifting, and walking.

Regarding the ergonomic benefits, the reviewed studies can be divided into three categories. The first category includes those in which the use of exoskeletons resulted in ergonomic improvements. The second category encompasses studies where no significant ergonomic improvements were observed. Lastly, the third category comprises studies that reported improvements but also highlighted certain constraints associated with the use of this type of assistive technology.

**Figure 6:**
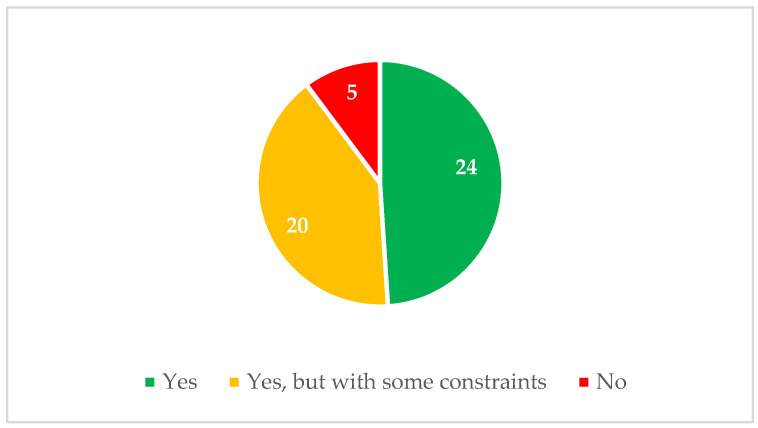
Distribution of the reviewed studies on ergonomic benefits.

The results presented in Figure 6 show the distribution of the studies within the above categories presented. Specifically, 24 studies [11,15,16,17,20,21,22,24,25,43,46,49,53,55,56,59,62,64,66,67,69,71,72,73] reported clear ergonomic improvements following the use of exoskeletons. However, 20 studies [9,10,14,18,23,42,44,48,51,52,57,58,61,63,65,68,70,75,76,86] indicated that while there were improvements, they were accompanied by certain constraints, likely reflecting limitations in the technology or specific challenges in its application. A smaller number, of five studies [45,47,54,60,74], did not observe any ergonomic benefits at all.

This distribution highlights that while most studies (44 out of 49) identified some level of ergonomic improvement, a significant proportion of these studies raised concerns or limitations that warrant further investigation. The small percentage of studies reporting no improvements suggests that while the potential for ergonomic benefits exists, it may not be universally applicable across all scenarios or populations.

The overall conclusions of the reviewed studies will be outlined below:

- **Effectiveness in reducing muscle activity and physical strain**: The majority of the studies demonstrate that exoskeletons—whether for the upper body, back, or lower limbs—significantly reduce muscle activity and physical strain during specific tasks. For example, studies such as those by Yin et al. (2021) [22] and Kong et al. (2021) [23] show reductions in upper-limb muscle activity, while Lazzaroni et al. (2020) [24] and Ding et al. (2023) [25] highlight the reduction in lumbar load during manual lifting tasks;
- **Constraints and trade-offs**: Although exoskeletons often reduce muscle fatigue and improve ergonomics, many studies report trade-offs in comfort, range of motion, and usability. For instance, Perez Luque et al. (2020) [9] and Cardoso et al. (2020) [76] indicate that while exoskeletons help to reduce strain, they also impose constraints on the range of motion and increase discomfort during prolonged use. This suggests that while beneficial for specific tasks, the long-term comfort and adaptability of these devices remain a challenge;
- **Task-specific benefits**: Several studies underscore the importance of selecting exoskeletons based on the specific demands of the task. For example, Kong et al. (2021) [23] and Schwartz et al. (2021) [45] show that passive exoskeletons provide significant benefits for overhead tasks but can increase strain during tasks performed at higher or lower heights. Shim et al. (2023) [58] and Schrøder Jakobsen et al. (2024) [69] highlight how the efficacy of these devices depends on task-specific requirements such as task height and lifting technique;
- **Active vs. passive exoskeletons**: Some studies compare active and passive exoskeletons, revealing that active systems often provide more substantial reductions in muscle activity but may hinder mobility or task performance, particularly during dynamic tasks. For instance, Schwartz et al. (2023) [68] report that active exoskeletons provide greater reductions in trunk muscle activity but may alter trunk kinematics and affect performance in dynamic environments;
- **User experience and ergonomics**: Several studies emphasize the need for improvements in the design and user experience of exoskeletons. Govaerts et al. (2024) [74] and Rafique et al. (2024) [10] suggest that while exoskeletons can reduce physical strain, there is a need for better design to enhance comfort, usability, and biomechanical compatibility for long-term use. This includes optimizing factors like device-to-body forces and ensuring that the exoskeleton does not interfere with the natural movement patterns of workers.

Globally, exoskeletons are a promising solution for reducing WMSD risks and improving ergonomics in physically demanding tasks. However, their effectiveness depends on task-specific factors, and improvements in comfort, adaptability, and long-term usability are needed to maximize their potential benefits across a broader range of applications.

## 4. Conclusions

This review provides a comprehensive overview of the methodologies and findings related to the evaluation of occupational exoskeletons, with a particular focus on WMSD risk assessment methods. Several key findings emerged from the analysis, offering valuable insights for future research, practical application, and design optimization.

### 4.1. Key Findings

The majority of studies reviewed were published between 2022 and 2024, with 10 participants being the most common sample size of the reviewed studies. Laboratory-based studies dominated the research landscape, with passive exoskeletons being the most frequently tested. Notably, the Paexo and Cray X exoskeletons were the most studied models. Regarding the supported body part, the back and upper arms were the most commonly targeted segments by the exoskeletons examined in the studies. These findings may correlate with the types of tasks tested since overhead and lifting tasks were the most commonly examined.

The review of the studies highlights the diverse methodologies and tools employed in evaluating occupational exoskeletons. Direct measurement techniques such as EMG and kinematic assessments were the most frequently used, while observational methods were underrepresented. Self-report methods and checklists, notably the Borg CR-10 Scale and the LPD, were commonly employed. Overall, the majority of studies reported benefits in reducing WMSD risk factors, although some highlighted limitations such as incompatibility between the exoskeleton and the task, discomfort due to contact points, restricted range of motion, and usability concerns.

The findings provide a foundational understanding that can guide future research, ensuring that exoskeletons are evaluated comprehensively, with considerations for both objective and subjective outcomes. Additionally, the main findings of each study are also presented. This review sets the stage for further exploration into optimizing the design and implementation of exoskeletons in occupational settings.

### 4.2. Implications for Practice

The findings suggest that exoskeletons have the potential to significantly reduce the WMSD risk factors. However, the effectiveness of these devices is influenced by several factors, including the specific occupational task, exoskeleton design, and user comfort. The high prevalence of passive exoskeletons in the studies indicates that they are simpler to implement but may not always be sufficient for dynamic, high-demand tasks. In contrast, active exoskeletons, while more complex, could offer greater support for these tasks. These insights are critical for designing exoskeletons that are better suited to the diverse demands of various industries.

Globally, this review highlights the need for more real-world evaluations of exoskeleton performance to better understand their practical benefits and limitations, with the ultimate goal of guiding future research and informing the development of more effective ergonomic interventions.

### 4.3. Limitations

This study has certain limitations that should be acknowledged. First, a pre-registered protocol detailing the main methodological features of this systematic review was not submitted to platforms such as PROSPERO. The absence of such prior registration may have introduced potential biases in the review process and highlights an area for improvement in future research to enhance methodological rigor. Second, the search strategy was based solely on the terms “Exoskeleton” and “WMSD”, chosen for being the most widely recognized in the literature. While the number of articles retrieved appears sufficiently representative to meet the study’s objectives, we recognize that a more extensive and detailed search strategy could improve the comprehensiveness of the results. This limitation offers a valuable opportunity for refinement in future research. Finally, a limitation of this study is the absence of a formal risk of bias assessment for the included articles. While the studies were qualitatively evaluated, a systematic and standardized approach to assessing the risk of bias was not employed. Future systematic reviews should incorporate a formal risk of bias assessment to enhance the transparency and robustness of their methodology.

### 4.4. Future Work

Future research should prioritize expanding real-world assessments to better understand the practical benefits and limitations of exoskeletons in diverse occupational settings. Particular attention should be given to active exoskeletons, as their application in occupational contexts remains in an early developmental stage, and there is a notable lack of studies exploring their potential in these environments. Additionally, addressing the ergonomic challenges and discomfort reported in some studies will be crucial in improving user acceptance and long-term efficacy. Building on these insights, a key direction for future work is the development of a comprehensive framework for evaluating occupational exoskeletons using the ergonomic assessment methodologies identified in this review. This framework would consolidate the most effective approaches, including direct measurement and self-report methods, to provide a standardized and practical tool for assessing exoskeletons in various occupational settings. By integrating findings from both laboratory-based evaluations and real-world applications, the framework aims to enhance the reliability and applicability of assessment results. Collaboration with industry stakeholders will also be essential to ensure the framework’s adaptability to diverse workplace contexts and to support the design of more effective and user-friendly exoskeletons.

The development of more effective exoskeletons has the potential to revolutionize occupational health and safety policies, particularly in industries where musculoskeletal disorders are prevalent. Improved exoskeleton designs could lead to safer working conditions, reducing injury rates and enhancing workforce productivity. As the evidence grows, these devices may become integral to workplace ergonomics strategies, driving the future of workplace safety standards and policies.

## Figures and Tables

**Table 2:** Key characteristics of the exoskeletons presented in the reviewed studies.

Authors (Year)	Exoskeleton	Actuation	Supported Body Part
Moyon et al. (2018) [42]	Pad	Passive	Upper arm
Schmalz et al. (2019) [43]	Paexo	Passive	Upper arm
Perez Luque et al. (2020) [9]	EksoEvo Ottobock’s Paexo Comau Mate	Passive	Upper arm
Alabdulkarim et al. (2020) [44]	(b–e) assistive device	Passive	Back and upper arm
Cardoso et al. (2020) [76]	Laevo v2	Passive	Back
Lazzaroni et al. (2020) [24]	XoTrunk	Active	Back
Kong et al. (2021) [23]	Cex	Passive	Legs
Schwartz et al. (2021) [45]	Corfor V2 Laevo V1	Passive	Back
Lazzaroni et al. (2021) [46]	XoTrunk	Active	Back
Yin et al. (2021) [22]	PUES_PGBA	Passive	Upper arm
Weston et al. (2022) [47]	EksoVest Airframe Shoulder X	Passive	Upper arm
vam der Have et al. (2022) [48]	Shoulder exoskeleton Prototype	Passive	Upper arm
Kong et al. (2022) [49]	Cex	Passive	Legs
Iranzo et al. (2022) [50]	Laevo v2	Passive	Back
Latella et al. (2022) [11]	Paexo	Passive	Upper arm
De Bock et al. (2022) [51]	Shoulder exoskeleton prototype (Exo4Work)	Passive	Upper arm
Goršič et al. (2022) [52]	Auxivo LiftSuit V1.1	Passive	Back
Sierotowicz et al. (2022) [53]	Paexo	Passive	Upper arm
Mitterlehner et al. (2023) [54]	Paexo	Passive	Back
Van Sluijs et al. (2023) [55]	Auxivo LiftSuit v2.0	Passive	Back
Garosi et al. (2023) [18]	Head/neck supporting exoskeleton	Passive	Head and neck
Walter et al. (2023) [56]	Cray X	Active	Back
Kong et al. (2023) [57]	Vex AirFrame	Passive	Upper arm
Shim et al. (2023) [58]	Cex Chairless Chair	Passive	Legs
Brunner et al. (2023) [59]	DeltaSuit prototype	Passive	Upper arm
Öçal et al. (2023) [21]	Exoskeleton prototype	Passive	Upper arm
Schrøder Jakobsen et al. (2023) [61]	Shoulder X V3	Passive	Upper arm
Govaerts et al. (2023) [60]	Paexo CrayX	Passive Active	Back
Verdel et al. (2023) [62]	Able	Active	Upper arm
Reimeir et al. (2023) [63]	Rakunie N-Ippin BionicBack hTRIUS SoftExo Lift Hunic Japet.W Japet Cray X	Passive Passive Passive Active Active	Back
van Sluijs et al. (2023) [16]	Auxivo LiftSuit v2.0	Passive	Back
Park et al. (2023) [17]	Elbow-sideWINDER	Active	Elbow
Ding et al. (2023) [25]	SE prototype	Passive	Back
Cuttilan et al. (2023) [64]	AirLevate	Passive	Back
De Bock et al. (2023) [65]	Shoulder exoskeleton prototype (Exo4Work)	Passive	Upper arm
Bhardwaj et al. (2023) [66]	BASE emulator exoskleton prototype	Passive	Back
Thang (2023) [67]	Passive exoskeleton with a mechanical arm	Passive	Upper arm
Schwartz et al. (2023) [68]	BackX Exoback V1 Cray X	Passive Active Active	Back
Musso et al. (2024) [14]	Skelex 360	Passive	Upper arm
Schrøder Jakobsen et al. (2024) [69]	BackX	Passive	Back
Rafique et al. (2024) [10]	EksoVest SuitX	Passive	Upper arm Legs
Davoudi Kakhki et al. (2024) [70]	BackX	Passive	Back
van Sluijs et al. (2024) [71]	OmniSuit	Passive	Back and upper arm
Favennec et al. (2024) [72]	Corfor	Passive	Back
Lee et al. (2024) [73]	Shoulder exoskeleton prototype	Passive	Upper arm
Gräf et al. (2024) [15]	Skelex 360	Passive	Upper arm
Govaerts et al. (2024) [74]	Paexo CrayX	Passive Active	Back
Bär et al. (2024) [75]	Laevo V2	Passive	Back
Cardoso et al. (2024) [20]	Auxivo lift suit V2 BionicBack hTRIUS	Passive	Back

**Table 3:** Methodological approach in the reviewed studies.

Authors (Year)	Tasks Assessed	WMSD Risk Assessment Methods Applied			
		Direct Measurement		Observational Methods	Self-Reports and Checklists
		Biomechanical	Physiological		
Moyon et al. (2018) [42]	Overhead tasks	N.A.	Heart rate	N.A.	Borg CR10
Schmalz et al. (2019) [43]	Overhead tasks	Kinematics: 3D motion capture system	Oxygen rate; unilateral EMG: AD, MD, PD, UT, MT, LT, BB, AS, LD, EOA muscles	N.A.	N.A.
Perez Luque et al. (2020) [9]	Overhead tasks	Kinematics: IMU tracking system	N.A.	N.A.	Subjective ratings questionnaire
Alabdulkarim et al. (2020) [44]	Push and pulling tasks-Light (15%) and Heavy (30%) of the participant weight	N.A.	Unilateral EMG: FDP, BB, AD, ES	N.A.	Borg CR10
Cardoso et al. (2020) [76]	Lifting tasks (~2 kg)	N.A.	Bilateral EMG: UT, ESI, ESLL	N.A.	Borg CR10, LPD and VAS
Lazzaroni et al. (2020) [24]	Lifting tasks (7.5–15 kg)	Kinematics: 3D motion capture system	Bilateral EMG: RA, EOA, IOA, ESLL, ESLT, ESI muscles	N.A.	N.A.
Kong et al. (2021) [23]	Task on lower height	N.A.	Unilateral EMG: UT, ES, MD, TB, BB, BF, RF, TA muscles	N.A.	Borg CR10
Schwartz et al. (2021) [45]	Lifting tasks (8 kg)	Kinematics: IMU tracking system	Bilateral EMG: ES muscle	N.A.	N.A.
Lazzaroni et al. (2021) [46]	Pulling tasks (10 kg–20 kg)	N.A.	Bilateral EMG: ESI, ESLL muscles	N.A.	VAS
Yin et al. (2021) [22]	Overhead tasks	N.A.	Unilateral EMG: AD, BB, TB, BR muscles	N.A.	N.A.
Weston et al. (2022) [47]	Overhead tasks	Kinematics: 3D motion capture system	Bilateral EMG: ESLL, IOA, LD, EOA, RA muscles	N.A.	Subjective ratings questionnaire
vam der Have et al. (2022) [48]	Lifting tasks (10 kg) and overhead tasks	Kinematics: 3D motion capture system	Unilateral EMG: LT, AD, MD, BB muscles	N.A.	N.A.
Kong et al. (2022) [49]	Task on lower height	Kinematics: IMU tracking system; Force: dynamometer	Unilateral EMG: MD, BB, TB, UT, ESLL, RF, TA, BF muscles	N.A.	N.A.
Iranzo et al. (2022) [50]	Lifting tasks (7–9 kg)	Kinematics: IMU tracking system	Unilateral EMG; ES, GMD, QF, STD muscles	N.A.	N.A.
Latella et al. (2022) [11]	Overhead tasks	Kinematics: IMU tracking system; Force: sensorized shoes	N.A.	N.A.	N.A.
De Bock et al. (2022) [51]	Lifting tasks (5 kg), overhead task and walking	N.A.	Bilateral EMG: AD, BB, TB, UT, ESLL, PM, LD muscles; heart rate; respiration	N.A.	LPD
Goršič et al. (2022) [52]	Lifting tasks (6.8–13.6 kg) and forward leaning tasks	Kinematics: 3D motion capture system	Bilateral EMG: ES, RA, MT muscles	N.A.	LPD
Sierotowicz et al. (2022) [53]	Overhead tasks	N.A.	Unilateral EMG: AD, MD, PD muscles	N.A.	N.A.
Mitterlehner et al. (2023) [54]	lifting, holding, carrying, bending, pushing, pulling, and walking tasks	N.A.	Heart rate	N.A.	NMQ and VAS
R. M. Van Sluijs et al. (2023) [55]	Forward leaning task (11 kg)	Kinematics: IMU tracking system	Unilateral EMG: ESLT, ESLL, QL, GM muscles	N.A.	N.A.
Garosi et al. (2023) [18]	Overhead tasks	N.A.	Bilateral EMG: SCM, SC, UT, AD muscles	N.A.	N.A.
Walter et al. (2023) [56]	Lifting tasks (15 kg)	N.A.	Bilateral EMG: ESL muscle	N.A.	RPE20
Kong et al. (2023) [57]	Overhead tasks	N.A.	Unilateral EMG: UT, MD, BB, TB, ES, RF, BF, TA muscles	N.A.	Borg CR10 and LPD
Shim et al. (2023) [58]	Task on lower height	N.A.	Unilateral EMG UT, MD, TB, BB, ES, RF, BF, TA muscles	N.A.	LPD
Brunner et al. (2023) [59]	Lifting tasks and overhead tasks	N.A.	Heart Rate; Unilateral EMG: BB, AD, MD, TB, PD, LT, LD, ES, RA muscles	N.A.	Borg CR10 and LPD
Öçal et al. (2023) [21]	Overhead tasks	N.A.	Unilateral EMG: TB, BB, PM, AD, MD, PD, UT, MT, LT, LD muscles	N.A.	N.A.
Schrøder Jakobsen et al. (2023) [61]	Lifting tasks (4.6–17.6 kg) and overhead tasks	Kinematics: 3D motion capture system	Bilateral EMG: ESLL, UT, AD muscles	N.A.	LPD and QUEAD
Govaerts et al. (2023) [60]	Lifting tasks (7 kg), walking, and range of motion movements	Kinematics: 3D motion capture system	N.A.	N.A.	LPD
Verdel et al. (2023) [62]	Reaching tasks	Kinematics: 3D motion capture system	Unilateral EMG: BR, BB, TB, AD, PD muscles	N.A.	Subjective ratings questionnaire
Reimeir et al. (2023) [63]	Lifting tasks (13 kg)	Kinematics: IMU tracking system	Unilateral EMG: LD, ES, MF, EOA muscles	N.A.	N.A.
R.M. van Sluijs et al. (2023) [16]	Forward leaning task (6–20 kg)	Kinematics: 3D motion capture system	Unilateral EMG: ESLL, ESLT, QL, GM, BF, STD, RA muscles	N.A.	Likert scale
Park et al. (2023) [17]	Lifting tasks (2–7 kg)	Kinematics: IMU tracking system	Unilateral EMG: BB, TB muscles	N.A.	N.A.
Ding et al. (2023) [25]	Lifting tasks (10 kg) and walking	N.A.	Unilateral EMG: ESI, ESLL, RF, BF muscles	N.A.	N.A.
Cuttilan et al. (2023) [64]	Lifting tasks (5 kg–20 kg)	Kinematics: 3D motion capture system	Bilateral EMG: ESI, ESLT muscles	N.A.	Subjective ratings questionnaire
De Bock et al. (2023) [65]	Lifting tasks and overhead tasks	Kinematics: 3D motion capture system	Unilateral EMG: ESLT, ESLL, QL, GM, BF, STD, RA muscles	N.A.	N.A.
Bhardwaj et al. (2023) [66]	Lifting tasks (20% of the participant’s body weight)	N.A.	Bilateral EMG: MF, ESLL, LD, RA muscles	N.A.	LPD and VAS
Thang (2023) [67]	Overhead tasks	Kinematics: IMU tracking system	Bilateral EMG: ESLL, LT, UT muscles	N.A.	Borg CR10 and Likert scale
Schwartz et al. (2023) [68]	Lifting tasks (10 kg) and overhead tasks	Kinematics: IMU tracking system	Bilateral EMG: BF, GM; ES, LD, LT muscles	N.A.	N.A.
Musso et al. (2024) [14]	Lifting tasks (10 kg) andoverhead tasks	N.A.	Bilateral EMG: SCM, SC, UT, AD muscles	N.A.	N.A.
Schrøder Jakobsen et al. (2024) [69]	Lifting tasks	Kinematics: IMU tracking system	Bilateral EMG: ESL, LT, UT muscles	N.A.	Borg CR10 Likert scale and QUEAD
Rafique et al. (2024) [10]	Lifting tasks (5–10 kg) and overhead tasks	N.A.	EMG: thighs and calf muscles	N.A.	N.A.
Davoudi Kakhki et al. (2024) [70]	Lifting tasks (7 kg), carrying task and walking	N.A.	N.A.	N.A.	Borg CR10 and subjective ratings questionnaire
van Sluijs et al. (2024) [71]	Lifting tasks and overhead tasks	N.A.	Unilateral EMG: MD, AD, LT, ESLL, ESI, ESLT, RA, GM muscles	N.A.	Likert scale
Favennec et al. (2024) [72]	Lifting tasks (10 kg) and overhead tasks	Kinematics: 3D motion capture system	Unilateral EMG: GM. TFL, EOA, RA, ES muscles	N.A.	Likert scale
Lee et al. (2024) [73]	Lifting tasks (5 kg) and overhead tasks	N.A.	Unilateral EMG: AD, MD, PD, UT, ES muscles	N.A.	N.A.
Gräf et al. (2024) [15]	Overhead tasks	N.A.	Unilateral EMG: ESLL, AD, LT muscles	N.A.	N.A.
Govaerts et al. (2024) [74]	Lifting, holding, carrying, bending, pushing, pulling, and walking tasks	N.A.	N.A.	N.A.	VAS
Bär et al. (2024) [75]	Forward leaning tasks	Kinematics: dimensional gravimetric position sensors	Unilateral EMG: ES, BF, RA, VL, GTM, LT muscles; Heart Rate	N.A.	N.A.
Cardoso et al. (2024) [20]	Lifting tasks (4 kg)	N.A.	Bilateral EMG: ESI, ESLL, RA muscles	REBA	Borg CR10

Surface electromyography (EMG); Not Available (N.A.); muscles: sternocleidomastoid (SCM); splenius capitis (SC); erector spinae longissimus lumborum (ESLL); erector spinae longissimus thoracis (ESLT); erector spinae iliocostalis (ESI); biceps branchii (BB); triceps branchii (TB); erector spinae (ES); rectus femoris (RF); biceps femoris (BF); tibialis anterior (TA); anterior deltoid (AD); middle deltoid (MD); posterior deltoid (PD); upper trapezius (UT); middle trapezius (MT); lower trapezius (LT); anterior serratus (AS); latissimus dorsi (LD); external obliquus abdominis (EOA); internal obliquus abdominis (IOA); rectus abdominis (RA); pectoralis major (PM); gluteus maximus (GM); gluteus medius (GMD); semitendinosus (STD); quadriceps femoris (QF); tensor fascia latae (TFL); brachioradialis (BR); multifidus (MF); quadratus lumborum (QL); grastocnemius medialis (GTM); vastus lateralis (VL); flexor digitorum profundus (FDP). Observational methods: Rapid Entire Body Assessment (REBA). Self-reports and checklists: Ratio Perceived Exertion 20 (RPE-20); Borg Category Ratio-10 Scale (Borg CR-10); Local Perceived Discomfort (LPD); Visual Analog Scale (VAS); Nordic Musculoskeletal Questionnaire (NMQ); Questionnaire for the Evaluation of Physical Assistive Devices (QUEAD).

**Table 4:** Synthesis of the reviewed studies focused on the objective and main conclusions.

Authors (Year)	Objective	Main Conclusions
Moyon et al. (2018) [42]	To evaluate the physical impact of a passive exoskeleton on manual sanding operations.	The use of the exoskeleton significantly reduced cardiac cost and physical workload during sanding tasks, especially in postures with hands above shoulder level, improving overall ergonomics and reducing fatigue. However, users also noted areas for improvement in comfort and usability for longer-term use.
Schmalz et al. (2019) [43]	To evaluate the biomechanical and metabolic effectiveness of a passive exoskeleton in supporting overhead work and reducing the physiological and musculoskeletal strain on workers.	The exoskeleton significantly reduces muscle activity in the shoulder and upper arm, as well as heart rate and oxygen consumption, thereby alleviating the physical burden during overhead tasks, while not imposing unnatural movement patterns, making it a promising solution for reducing WMSD in overhead work.
Perez Luque et al. (2020) [9]	To evaluate and compare how the use of three different passive upper body exoskeletons affects the range of motion of workers during overhead manual assembly tasks.	The tested exoskeletons can effectively reduce the risk of musculoskeletal disorders in overhead tasks, different models impose varying limitations on range of motion, and further development is needed to minimize these limitations and improve overall comfort and usability for a broader range of tasks.
Alabdulkarim et al. (2020) [44]	To develop a low-cost exoskeleton designed to assist with anterior load carriage tasks and to evaluate its effectiveness in reducing physical demands during walking tasks with different load weights.	The exoskeleton significantly reduced muscle activity, while slightly increasing perceived discomfort in the lower back.
Cardoso et al. (2020) [76]	To assess the effects of a passive back-support exoskeleton on muscle activity and discomfort during industrial tasks.	The exoskeleton provides back support and reduces muscle activity by 0.8% to 3.8%; it also limits movement, interferes with task performance, and increases discomfort in various body regions, suggesting that it may not be well-suited for tasks requiring a wide range of motions.
Lazzaroni et al. (2020) [24]	To investigate the effects of an acceleration-based assistive strategy for controlling a back-support exoskeleton during manual lifting tasks, focusing on spine kinematics, muscle activation, and lumbar compression.	The exoskeleton reduced peak compression force on the L5S1 disk by up to 16%, with all control strategies showing similar effectiveness in reducing lumbar load.
Kong et al. (2021) [23]	To establish ergonomic guidelines for the use of a passive lower-limb exoskeleton by evaluating muscle activities and discomfort levels at various working heights.	The exoskeleton significantly reduces muscle activity and discomfort at working heights below 100 cm, making it beneficial for tasks performed at low heights, while its effectiveness diminishes and even increases discomfort and muscle activity at higher working heights (100–140 cm).
Schwartz et al. (2021) [45]	To compare the effectiveness of soft versus rigid back-support exoskeletons in reducing ES muscle activity during a dynamic lifting and lowering task, considering different trunk sagittal inclinations.	The soft exoskeleton significantly reduced ES muscle activity (by up to 11.1%) across most of the trunk inclinations, while the rigid exoskeleton showed minimal reductions and even increased muscle activity in certain positions, particularly during deep trunk flexion, suggesting that the soft design may offer better overall support during lifting tasks.
Lazzaroni et al. (2021) [46]	To design and evaluate a control strategy for an active back-support exoskeleton to assist with pulling tasks.	The exoskeleton control strategy significantly reduced spinal muscle activity (up to 38% reduction in mean activation) during pulling tasks, with positive subjective feedback regarding comfort, ease of use, and perceived assistance, indicating the strategy’s effectiveness in mitigating low back pain risks associated with pulling activities.
Yin et al. (2021) [22]	To design and test a passive upper-arm exoskeleton to reduce muscle effort in the upper limbs during repetitive manual tasks.	The exoskeleton significantly reduced muscle fatigue, particularly in the biceps brachii (up to 67.8%), and improved efficiency during both static and dynamic overhead tasks, demonstrating its effectiveness in reducing upper limb strain and the risk of WMSD.
Weston et al. (2022) [47]	To evaluate the physiological and biomechanical effects of three passive upper-arm exoskeletons during simulated overhead work.	The exoskeletons provided little to no significant physiological benefit in terms of tissue oxygenation during the tasks, with only one instance of statistically significant improvement. No significant increases in spinal loading or discomfort were observed, suggesting that while the exoskeletons do not substantially reduce fatigue, they also do not introduce additional strain.
vam der Have et al. (2022) [48]	To evaluate the Exo4Work shoulder exoskeleton’s effectiveness in reducing muscle and joint loading during simulated occupational tasks performed above shoulder height, and to examine its impact on neighboring joints.	The Exo4Work exoskeleton significantly reduces musculoskeletal loading in the shoulder and elbow during overhead tasks without increasing the load on the lower back, hip, or knee, but may increase loading during tasks performed below shoulder height, indicating its suitability primarily for overhead work.
Kong et al. (2022) [49]	To assess the reduction in muscle loads during bolting tasks when using a lower-limb passive exoskeleton.	Wearing the lower-limb exoskeleton significantly reduces muscle activity in the lower extremities (up to 82.4–89.4%), indicating its effectiveness in alleviating physical strain during bolting tasks, especially at lower working heights.
Iranzo et al. (2022) [50]	To evaluate the effectiveness of a passive lumbar exoskeleton in reducing muscle activity and fatigue during manual material handling tasks.	The exoskeleton significantly reduces muscle activity (8% to 10.2%). It also decreases fatigue, particularly in the STD muscle, while causing only minimal restrictions on the range of motion, demonstrating its potential to alleviate physical strain in tasks involving manual material handling.
Latella et al. (2022) [11]	To analyze the effects of using a passive upper-arm exoskeleton on whole-body joint torques during overhead work, aiming to assess the reduction in internal biomechanical loads on various body regions.	The exoskeleton significantly reduces the joint effort in the shoulders and torso (up to 86% reduction), but this effort is partially transferred to the lower limbs, particularly the hips and thighs, highlighting both the benefits and redistribution of mechanical loads across the body.
De Bock et al. (2022) [51]	To assess the effects of a passive shoulder exoskeleton on muscle activity, muscle fatigue, and subjective experiences during overhead and non-overhead industrial tasks.	The exoskeleton significantly reduces muscle activity and fatigue in the anterior deltoid (up to 16% reduction in muscle activity and 41% reduction in fatigue) during isometric overhead work. However, it minimally impacts dynamic tasks and can increase discomfort at body-exoskeleton contact points.
Goršič et al. (2022) [52]	To evaluate the short-term effects of passive back support exoskeleton on muscle activity and user comfort during lifting and static leaning task.	The reduced electromyographic activity in the ES and MT muscles during both lifting and leaning tasks. However, it was found to be suboptimal for long-term use, as participants reported mild to moderate discomfort, particularly around the hips, due to the stiffness of the exoskeleton design.
Sierotowicz et al. (2022) [53]	To assess the effectiveness of an adaptive industrial exoskeleton using force myography to adjust support levels in real-time during overhead work.	The adaptive control system successfully reduced shoulder muscle activity by up to 31% during lifting tasks by adjusting the level of assistance based on the weight lifted, leading to improved shoulder stability and reduced strain without compromising user comfort or range of motion.
Mitterlehner et al. (2023) [54]	To evaluate both the objective and subjective effects of a passive low-back exoskeleton during simulated logistics tasks, focusing on heart rate, trunk kinematics, throughput, and user experience.	While the exoskeleton showed minimal impact on heart rate and throughput, it significantly reduced trunk acceleration and inclination in certain tasks, although users reported moderate discomfort and restrictions in movement, suggesting the need for task-specific adaptations and improvements in usability.
R. M. Van Sluijs et al. (2023) [55]	To develop a method to quantify the reduction in back and hip muscle fatigue provided by lift-support exoskeletons during static forward-leaning task.	The exoskeleton significantly reduced muscle activity in the ESLT and ESLL muscles (by 33% and 13.2%, respectively) and the GM muscle (16.3%). Additionally, the exoskeleton delayed muscle fatigue, particularly in the GM and QL muscles, demonstrating its effectiveness in reducing physical strain during lifting tasks.
Garosi et al. (2023) [18]	To investigate the effects of a passive head/neck supporting exoskeleton on the electromyographic fatigue threshold of neck and shoulder muscles during repetitive overhead tasks.	The exoskeleton significantly delayed the onset of muscle fatigue in the sternocleidomastoid and trapezius muscles, suggesting its potential as an ergonomic intervention to reduce musculoskeletal risks, although further studies are needed to evaluate its broader application.
Walter et al. (2023) [56]	To examine how an active exoskeleton reduces erector spinae muscle activity during lifting tasks and to assess its impact on muscle activation and perceived exertion under different support levels.	The active exoskeleton significantly reduces ES muscle activity and perceived exertion during lifting tasks, with greater reductions observed as the support level increases, indicating the exoskeleton’s potential to lower physical strain and mitigate the risk of musculoskeletal disorders.
Kong et al. (2023) [57]	To evaluate the efficacy of two passive upper-arm exoskeletons in reducing musculoskeletal load and muscle activity during overhead tasks in industrial settings.	The passive upper-arm exoskeletons significantly reduce muscle activity in the upper limb muscles, indicating their potential as an effective intervention for reducing physical exertion and WMSD risks during overhead tasks, although the effects on subjective discomfort were minimal.
Shim et al. (2023) [58]	To evaluate the effects of passive lower-limb exoskeletons on muscle activity during tasks performed at various working heights, focusing on both upper and lower-limb muscle responses and discomfort levels.	Passive lower-limb exoskeletons significantly reduce muscle activity in the lower limbs, particularly at lower working heights, while increasing upper-limb muscle activity at higher working heights, suggesting that the use of these exoskeletons is more beneficial at working heights of 85 cm or lower to optimize ergonomic benefits.
Brunner et al. (2023) [59]	To evaluate the effect of a passive shoulder exoskeleton, on muscle activity, cardiac cost, and perceived exertion during overhead tasks, aiming to reduce the physical strain associated with WMSD.	Significantly reduced muscle activity (up to 64% in the deltoideus medius), cardiac cost (15%), and perceived exertion (21.5%) during overhead tasks, without increasing the load on the lower back or abdomen, demonstrating its potential as an effective intervention for workers at high WMSD risk.
Öçal et al. (2023) [21]	To develop an innovative passive upper-arm exoskeleton and investigate its effects on muscle activity during overhead and extended forearm tasks.	The exoskeleton significantly reduces muscle activity, with up to a 55% reduction in the middle deltoid and 48% in the anterior deltoid during overhead tasks, showing its potential for improving worker comfort and reducing WMSD risk.
Schrøder Jakobsen et al. (2023) [61]	To evaluate the biomechanical changes, acceptance, and usability of a passive shoulder exoskeleton during manual material handling tasks in a logistics environment, focusing on muscle activity, perceived effort, and user feedback, after a five-week familiarization period.	The exoskeleton significantly reduced muscle activity in the AD (13–39%) and UT muscle (16–60%), along with a reduction in perceived effort. However, the familiarization period showed low adherence, and workers expressed decreased positive emotions towards the exoskeleton, raising concerns about its suitability for long-term use in logistics.
Govaerts et al. (2023) [60]	To compare the effects of an active and passive industrial back-support exoskeleton on physical work performance during simulated material handling tasks.	Both exoskeletons hindered physical work performance by increasing movement duration compared to no exoskeleton, particularly in tasks involving walking and trunk bending.
Verdel et al. (2023) [62]	To investigate the trade-off between mechanical complexity and interaction quality in upper-limb exoskeleton interfaces by analyzing the influence of passive rotations in the forearm interface during sagittal plane reaching movements.	Incorporating passive degrees of freedom in the forearm interface significantly improves interaction quality by reducing interaction forces and muscle activity without compromising kinematics, offering a promising design solution for balancing complexity and usability in exoskeletons.
Reimeir et al. (2023) [63]	To investigate the acute effects of five different back-support exoskeletons (active and passive), on trunk muscle activity and joint kinematics during a combined logistics task.	The exoskeletons significantly reduced muscle activity during lifting tasks, with passive exoskeletons showing a notable reduction in trunk flexion and task duration. However, the support provided by these devices varies depending on their functional mechanisms, highlighting the need for exoskeletons to be assessed according to their designed force paths to ensure long-term injury prevention.
R.M. van Sluijs et al. (2023) [16]	To evaluate the physiological benefits of a passive back-support exoskeleton during lifting tasks and forward-leaning postures.	The exoskeleton significantly reduces muscle activity in the back (up to 25.59% during forward leaning and 20.52% during lifting) without affecting leg and abdominal muscle activity or joint kinematics, indicating that the exoskeleton can alleviate physical strain during repetitive lifting and forward-leaning tasks.
Park et al. (2023) [17]	To design, control, and validate a novel elbow exoskeleton, aimed at assisting elbow flexion and extension during occupational tasks, while minimizing joint misalignment and discomfort.	The exoskeleton significantly reduces muscle activation in the BB and TB muscles (up to 38.8% and 37%, respectively, for a 2 kg load) without overly restricting elbow range of motion, making it a promising tool for reducing muscle strain during repetitive industrial tasks.
Ding et al. (2023) [25]	To develop and validate a novel passive back-support exoskeleton with a spring-cable-differential mechanism, designed to assist with lifting tasks while minimizing resistance during walking, to reduce the risk of lower back injuries.	The exoskeleton effectively reduces ES muscle activation by up to 41% during lifting tasks without significantly affecting leg and back muscle activity during walking, demonstrating its potential to alleviate lower back strain while maintaining comfort during movement.
Cuttilan et al. (2023) [64]	To investigate the effectiveness of a fabric-based, pneumatic exoskeleton in reducing lower-back muscle activation and discomfort during manual handling tasks	The exoskeleton significantly reduces muscle activation of the erector spinae at the L5 level (up to 35% during lifting tasks), without limiting the range of motion or increasing discomfort, making it a promising tool for reducing the risk of lower-back.
De Bock et al. (2023) [65]	To investigate how a passive shoulder exoskeleton mitigates the effects of physical fatigue on overhead work precision performance, muscle activity, and shoulder kinematics.	The exoskeleton reduces muscle activity in the anterior and medial deltoid muscles and mitigates fatigue-induced changes in shoulder joint kinematics, particularly reducing compensatory movements during overhead work. However, the exoskeleton did not significantly impact precision performance.
Bhardwaj et al. (2023) [66]	To investigate the effect of moment arm orientation in a passive back-assist exosuit on device-to-body forces, perceived discomfort, and muscle activity during lifting and lowering tasks.	The moment arm configuration significantly impacts the device-to-body forces, with the C4 configuration reducing forces at the shoulder and waist by up to 44.6% and 22.2%, respectively, while also minimizing perceived discomfort, demonstrating the importance of optimizing exoskeleton design for user comfort and effectiveness.
Thang (2023) [67]	To evaluate the effectiveness of a passive exoskeleton in reducing muscle strain and discomfort during overhead lifting tasks.	The passive exoskeleton significantly reduced muscle activity in the anterior and medial deltoid muscles by 30% to 80% across different payloads, with greater reductions observed at heavier loads. The exoskeleton also decreased discomfort in the shoulders and back, demonstrating its potential to reduce the WMSD risk during repetitive overhead tasks.
Schwartz et al. (2023) [68]	To evaluate the biomechanical consequences of using passive and active back-support exoskeletons during various manual handling tasks.	Both passive and active exoskeletons reduce trunk extensor muscle activity, but active exoskeletons provide greater reductions (up to 62%) compared to passive ones (up to 27%). Active exoskeletons also tend to alter trunk kinematics more significantly, particularly in dynamic tasks, highlighting the importance of task-specific exoskeleton selection.
Musso et al. (2024) [14]	To assess the impact of an upper-limb exoskeleton on muscle activity during various construction and manufacturing tasks, including overhead assembly, bricklaying, and box moving, to determine its potential in reducing.	The exoskeleton effectively reduces shoulder muscle activation during tasks performed above shoulder level, such as overhead assembly, but increases muscle activation for tasks below shoulder level, like bricklaying and box moving, indicating task-specific benefits and limitations that should be considered for ergonomic optimization.
Schrøder Jakobsen et al. (2024) [69]	To investigate how on-site training with a passive back exoskeleton affects the biomechanics of logistic workers, specifically focusing on muscle activity, joint kinematics, and the acceptance and comfort of the device after a 5-week training period.	The exoskeleton training optimized the interaction between the workers and the device, resulting in a 6–9% reduction in peak back muscle activity and a decrease in knee flexion, promoting a more stooped lifting technique, which suggests the benefits of incorporating training when implementing passive exoskeletons in logistics to reduce biomechanical load.
Rafique et al. (2024) [10]	To evaluate the benefits of passive exoskeletons in reducing muscle effort and preventing WMSD in workers performing physically demanding tasks, and to provide recommendations for design improvements.	The passive exoskeletons significantly reduce muscle activity (up to 66% for upper body tasks and up to 54% for lower body tasks), demonstrating their potential to alleviate the physical strain associated WMSD, although further design enhancements are necessary to improve user comfort and biomechanical compatibility.
Davoudi Kakhki et al. (2024) [70]	To evaluate the efficacy of a passive back-support exoskeleton in enhancing ergonomics and reducing physical discomfort during manual handling tasks.	The exoskeleton significantly reduced discomfort and physical exertion in the lower back, shoulders, and knees, demonstrating its potential to improve ergonomic posture and prevent musculoskeletal disorders, although some users reported constraints on movement and moderate physical effort while using the device.
van Sluijs et al. (2024) [71]	To present the design and evaluate the effectiveness of a passive multi-joint exoskeleton, in reducing muscle activity and supporting both back and shoulder during full-range vertical lifting tasks.	The exoskeleton significantly reduces muscle activity in the deltoid, trapezius, and ES muscles (by up to 75% for the shoulder and 31% for the back), demonstrating its potential to alleviate physical strain in occupations requiring dynamic movements across a large vertical range.
Favennec et al. (2024) [72]	To characterize the familiarization process with a soft back-support occupational exoskeleton and determine the time required for stabilizing biomechanical variables such as joint kinematics, postural stability, muscle activity, and performance during stoop and squat lifting tasks.	The familiarization process for the soft back-support exoskeleton leads to significant changes in thoracic kinematics, pressure perception, and performance, with stabilization occurring after three or four sessions. This suggests that workers need approximately four familiarization sessions, each lasting one hour, for their motor control and exoskeleton perception to stabilize, ensuring accurate long-term assessments of exoskeleton benefits.
Lee et al. (2024) [73]	To propose a novel passive shoulder exoskeleton using link chains and magnetic spring joints, aimed at supporting the upper arm during overhead work and reducing musculoskeletal strain in the shoulders.	The exoskeleton significantly reduced shoulder muscle activity (up to 30% for AD muscles during overhead tasks) while providing effective torque assistance without compromising range of motion, making it a promising solution for preventing shoulder injuries during repetitive overhead tasks.
Gräf et al. (2024) [15]	To evaluate the impact of a passive upper-body exoskeleton on muscle activity and precision during overhead single and dual tasks.	The exoskeleton significantly reduced muscle activity in the deltoideus and trapezius muscles, particularly under dual-task conditions, and improved precision after fatigue, highlighting its potential to support overhead work while reducing musculoskeletal strain without compromising task accuracy.
Govaerts et al. (2024) [74]	To compare the functional performance impacts, perceived task difficulty and general discomfort of active and passive industrial back exoskeletons during various work-related tasks.	The active exoskeleton significantly hindered work performance in multiple tasks (up to 22%), especially in dynamic activities such as walking and stair climbing, while the passive exoskeleton performed comparably to no exoskeleton in most tasks.
Bär et al. (2024) [75]	To evaluate the effects of using a passive back exoskeleton during a simulated sorting task that involves a static forward bent posture, focusing on its influence on muscle activity, posture, and heart rate.	The exoskeleton significantly reduced BF muscle activity (by 8.1%) and showed minor reductions in ES muscle activity (by 1.3%), with increased hip and knee flexion angles and a slight decrease in heart rate (by 2.1 bpm), suggesting that the exoskeleton primarily supports hip extension during tasks requiring a forward bent posture.
Cardoso et al. (2024) [20]	To assess the short-term effects of using dual passive back-support exoskeletons on WMSD risk factors in logistics operations.	Both exoskeletons reduced perceived exertion, especially during tasks involving trunk flexion, and improved posture during manual lifting tasks. The Htrius exoskeleton showed slightly better performance in reducing lumbar muscle activity and improving comfort, suggesting its potential for reducing WMSD risk in logistics operations.

## Data Availability

Not applicable.

## Appendix A

**Table A1:** Preferred Reporting Items for Systematic Reviews and Meta-Analyses Extension for Scoping Reviews (PRISMAScR) Checklist.

Section and Topic	Item #	Checklist Item	Reported on Page #
**Title**			
Title	1	Identify the report as a systematic review.	1
**Abstract**			
Abstract	2	See the PRISMA 2020 for Abstracts checklist.	1
**Introduction**			
Rationale	3	Describe the rationale for the review in the context of existing knowledge.	2
Objectives	4	Provide an explicit statement of the objective(s) or question(s) the review addresses.	3
**Methods**			
Eligibility criteria	5	Specify the inclusion and exclusion criteria for the review and how studies were grouped for the syntheses.	3–5
Information sources	6	Specify all databases, registers, websites, organisations, reference lists and other sources searched or consulted to identify studies. Specify the date when each source was last searched or consulted.	3
Search strategy	7	Present the full search strategies for all databases, registers and websites, including any filters and limits used.	3–5
Selection process	8	Specify the methods used to decide whether a study met the inclusion criteria of the review, including how many reviewers screened each record and each report retrieved, whether they worked independently, and if applicable, details of automation tools used in the process.	3–5
Data collection process	9	Specify the methods used to collect data from reports, including how many reviewers collected data from each report, whether they worked independently, any processes for obtaining or confirming data from study investigators, and if applicable, details of automation tools used in the process.	3–5
Data items	10a	List and define all outcomes for which data were sought. Specify whether all results that were compatible with each outcome domain in each study were sought (e.g., for all measures, time points, analyses), and if not, the methods used to decide which results to collect.	4
	10b	List and define all other variables for which data were sought (e.g., participant and intervention characteristics, funding sources). Describe any assumptions made about any missing or unclear information.	4
Study risk of bias assessment	11	Specify the methods used to assess risk of bias in the included studies, including details of the tool(s) used, how many reviewers assessed each study and whether they worked independently, and if applicable, details of automation tools used in the process.	28
Effect measures	12	Specify for each outcome the effect measure(s) (e.g., risk ratio, mean difference) used in the synthesis or presentation of results.	3–5
Synthesis methods	13a	Describe the processes used to decide which studies were eligible for each synthesis (e.g., tabulating the study intervention characteristics and comparing against the planned groups for each synthesis (item #5)).	3–5
	13b	Describe any methods required to prepare the data for presentation or synthesis, such as handling of missing summary statistics, or data conversions.	3–5
	13c	Describe any methods used to tabulate or visually display results of individual studies and syntheses.	3–5
	13d	Describe any methods used to synthesize results and provide a rationale for the choice(s). If meta-analysis was performed, describe the model(s), method(s) to identify the presence and extent of statistical heterogeneity, and software package(s) used.	3–5
	13e	Describe any methods used to explore possible causes of heterogeneity among study results (e.g., subgroup analysis, meta-regression).	-
	13f	Describe any sensitivity analyses conducted to assess robustness of the synthesized results.	-
Reporting bias assessment	14	Describe any methods used to assess risk of bias due to missing results in a synthesis (arising from reporting biases).	28
Certainty assessment	15	Describe any methods used to assess certainty (or confidence) in the body of evidence for an outcome.	-
**Results**			
Study selection	16a	Describe the results of the search and selection process, from the number of records identified in the search to the number of studies included in the review, ideally using a flow diagram.	3–5
	16b	Cite studies that might appear to meet the inclusion criteria, but which were excluded, and explain why they were excluded.	-
Study characteristics	17	Cite each included study and present its characteristics.	5–7
Risk of bias in studies	18	Present assessments of risk of bias for each included study.	28
Results of individual studies	19	For all outcomes, present, for each study: (a) summary statistics for each group (where appropriate) and (b) an effect estimate and its precision (e.g., confidence/credible interval), ideally using structured tables or plots.	5–7
Results of syntheses	20a	For each synthesis, briefly summarise the characteristics and risk of bias among contributing studies.	28
	20b	Present results of all statistical syntheses conducted. If meta-analysis was done, present for each the summary estimate and its precision (e.g., confidence/credible interval) and measures of statistical heterogeneity. If comparing groups, describe the direction of the effect.	-
	20c	Present results of all investigations of possible causes of heterogeneity among study results.	-
	20d	Present results of all sensitivity analyses conducted to assess the robustness of the synthesized results.	-
Reporting biases	21	Present assessments of risk of bias due to missing results (arising from reporting biases) for each synthesis assessed.	28
Certainty of evidence	22	Present assessments of certainty (or confidence) in the body of evidence for each outcome assessed.	-
**Discussion**			
Discussion	23a	Provide a general interpretation of the results in the context of other evidence.	27
	23b	Discuss any limitations of the evidence included in the review.	28
	23c	Discuss any limitations of the review processes used.	28
	23d	Discuss implications of the results for practice, policy, and future research.	27
**Other Information**			
Registration and protocol	24a	Provide registration information for the review, including register name and registration number, or state that the review was not registered.	28
	24b	Indicate where the review protocol can be accessed, or state that a protocol was not prepared.	28
	24c	Describe and explain any amendments to information provided at registration or in the protocol.	28
Support	25	Describe sources of financial or non-financial support for the review, and the role of the funders or sponsors in the review.	29
Competing interests	26	Declare any competing interests of review authors.	29
Availability of data, code and other materials	27	Report which of the following are publicly available and where they can be found: template data collection forms; data extracted from included studies; data used for all analyses; analytic code; any other materials used in the review.	-
